# Population-wide modelling reveals prospects of marker-assisted selection for parasitic mite resistance in honey bees

**DOI:** 10.1038/s41598-024-58596-5

**Published:** 2024-04-03

**Authors:** Regis Lefebre, Bart J. G. Broeckx, Lina De Smet, Luc Peelman, Dirk C. de Graaf

**Affiliations:** 1 Laboratory of Molecular Entomology and Bee Pathology (L-MEB), Department of Biochemistry and Microbiology, Faculty of Sciences, Ghent University, Ghent, Belgium; 2 Laboratory of Animal Genetics, Department of Veterinary and Biosciences, Faculty of Veterinary Medicine, Ghent University, Ghent, Belgium

**Keywords:** Animal breeding, Genetic association study, Genetic markers, Entomology

## Abstract

In 2019, a joint eight-variant model was published in which eight single nucleotide polymorphisms (SNPs) in seven *Apis mellifera* genes were associated with *Varroa destructor* drone brood resistance (DBR, i.e. mite non-reproduction in drone brood). As this model was derived from only one Darwinian Black Bee Box colony, it could not directly be applied on a population-overarching scale in the northern part of Belgium (Flanders), where beekeepers prefer the *carnica* subspecies. To determine whether these eight SNPs remained associated with the DBR trait on a Flemish colony-broad scope, we performed population-wide modelling through sampling of various *A. mellifera carnica* colonies, DBR scoring of *Varroa*-infested drone brood and variant genotyping. Novel eight-variant modelling was performed and the classification performance of the eight SNPs was evaluated. Besides, we built a reduced three-variant model retaining only three genetic variants and found that this model classified 76% of the phenotyped drones correctly. To examine the spread of beneficial alleles and predict the DBR probability distribution in Flanders, we determined the allelic frequencies of the three variants in 292 *A. mellifera carnica* queens. As such, this research reveals prospects of marker-assisted selection for *Varroa* drone brood resistance in honeybees.

## Introduction

From the end of the twentieth century on, the ectoparasitic mite *Varroa destructor* switched host from the Asian honey bee *Apis cerana* to the Western honey bee *Apis mellifera* by globalization of apiculture^1^. Due to a lack of co-evolution, the mite has since threatened the survival of *A. mellifera* colonies worldwide by impairing the brood, weakening adult bees’ capabilities and acting as a vector for several honey bee viruses^2–7^. As an obligate parasite, *V. destructor* can only reproduce in the honey bees’ brood and host sensing occurs predominantly pheromonically^2,8–12^. The mature mite is attracted by volatile semiochemicals expressed by the 4th and 5th instar larvae and prefers drone brood over worker brood as 5th instar drone larvae express higher levels of these chemicals compared to worker larvae^2,9,11–15^. Also non-chemical factors play crucial roles in the preference of the mite for drone brood, such as bigger cell size^16–18^ and brood temperature^19^. After cell invasion by *Varroa* and capping, the foundress mite may produce up to three mature fertilized daughters in worker cells^20,21^ and four in drone cells^20,22^ before emergence of the newborn bee. Higher numbers of viable offspring mites have repeatedly been reported upon emergence in drone brood compared to worker brood, despite equal percentages of non-reproducing mites^21–27^. As such, presence of drone brood in the colony may positively impact the *Varroa* population growth, increasing the risk for colony death^28^.

Already since the early spread of *Varroa* on the Western honey bee, numerous reports described the natural survival of untreated *A. mellifera* colonies under *V. destructor* mite infestations on all continents, except Antarctica (reviewed in^29,30^). In these colonies, various behavioral and individual resistance traits have been characterized that lower the *V. destructor* mite burden^29,30^. Cases of ‘suppressed mite reproduction’ (SMR) being a prominent defensive trait were reported in Brazil^31,32^, Sweden^33–35^, France^35,36^, Norway^37^ and South Africa^38,39^. SMR is nowadays defined as a heritable trait in which cell-invading *Varroa* mites fail to produce (mated) mature offspring, caused by *i.a.* yet unknown brood-related traits. Reported h^2^ values range from 0.18 up to 0.46, mainly dependent on the population or subspecies screened for heritability estimation^29,40–44^. For example, a h^2^ of 0.44 has been described in Carniolan bees (*Apis mellifera carnica*), which is the preferred subspecies of *A. mellifera* by beekeepers in Flanders (Belgium) due to its gentleness and productivity^43^. Current hypotheses state that brood-intrinsic SMR may be the result of derangements in the synchronized host-parasite signal interaction^45–47^. Colony-level SMR is currently scored by microscopically phenotyping capped worker- or drone brood for fecundity-based reduction in mite reproduction (number of offspring in relation to pupal age) and/or fertility-based reduction in mite reproduction (presence or absence of at least one offspring mite)^48–51^. Phenotyping the trait is however time-consuming and requires sufficient data points for accurate and precise scoring^48,52^. Consequently, brood-intrinsic SMR is limitedly implemented in selection- or breeding programs aiming to increase resilience towards *V. destructor*, strengthening a top-down selection approach in which a few beekeepers provide further breeding materials, thereby narrowing genetic diversity.

A possible solution for these shortcomings is the transition from traditional phenotyping to genotyping methods, aiming to breed with or reconstruct genetic profiles associated with the protective trait of interest (= marker-assisted selection or MAS). Quantitative trait loci (QTL) not only support scientific research to identify the molecular basis behind a quantitative trait, but also allow bottom-up selective breeding approaches based on only a few genetic markers^53^. For instance, cost-effective high-throughput genotyping of genetic markers associated with a trait of interest permits more candidate populations in the breeding program and widens the genetic diversity from which further breeding may be accomplished^54^. In *A. mellifera*, numerous QTL (mapping) analyses and associations have already been performed for *Varroa*-sensitive hygiene (VSH)^55–60^, grooming behavior^61^ and mite non-reproduction (MNR)^29,62–65^. The current study builds further on an earlier published joint eight-variant model, in which eight single nucleotide polymorphisms (SNPs) in seven different genes were associated with drone brood resistance (DBR, i.e. MNR in drone brood) in one hybrid Dutch *Varroa*-resistant/sensitive Darwinian Black Bee Box (DBBB) colony^66,67^. For the first time, this study reported the use of Whole Exome Sequencing (WES) for high resolution phenotype-associated variant detection in *A. mellifera*. More specifically, hybrid *Varroa*-resistant/sensitive (VR/VS) colonies were established by artificially crossing virgin *Varroa*-resistant (VR/VR) queens from The Netherlands (DBBB program) with local *Varroa*-sensitive (VS) drones through single drone insemination (SDI)^37,66,68,69^. The created hybrid VR/VS queens were then mated naturally, and their offspring drone brood was phenotyped for the DBR trait^66^. Only the VR/VS hybrid queen originating from the Amsterdam Water Dunes population in The Netherlands (DBBB) showed a percentage of non-reproducing mites in the drone brood that differed significantly from a local control strain^66^. Thirty-five out of 69 drones (51%) of this queen contained a single non-reproducing mother mite (DBR phenotype). Next, 32 DBR positive- and 32 DBR negative drones were subjected to Illumina WES analysis, single-marker Fisher exact tests and elastic net penalized regression^66^. The resulting joint eight-variant model, comprising six risk and two protective mutations, classified 56 of the 64 drone phenotypes correctly solely based on the genotypes of the drones (88%)^66^.

As this joint eight-variant model (together with its effect sizes) was derived from only one hybrid colony from the Amsterdam Water Dunes (The Netherlands)^66^, it was uncertain whether it held true in a different subspecies on a population-wide scale in the northern part of Belgium (Flanders), with varying genetics, environmental conditions and beekeeping practices. Therefore, we conducted a population-wide genotype–phenotype association study for the eight described SNPs through Flemish population-wide sampling of *Apis mellifera carnica* honey bee colonies, DBR scoring of *Varroa*-infested drone brood and variant genotyping of the phenotyped drone pupae using qPCR’s with dual-labelled probes. Novel eight-variant mixed-effect modelling was performed on the obtained genotype–phenotype data set with the eight variants as fixed effects and the beekeeper as random effect, and classification performance of the eight SNPs was evaluated by Receiver Operating Characteristic (ROC) curve construction. Besides, a reduced three-variant model was built containing only three of the eight genetic variants (significant in single variant tests) as fixed effects and the beekeeper as random effect, and classification performance was compared with the new eight-variant mixed-effect model. To determine the spread of beneficial alleles of the reduced three-variant model’s SNP markers in *A. mellifera carnica* queens from Flanders, we investigated the allelic frequencies by drone leg pooling and qPCR’s with dual-labelled probes. Using the reduced three-variant model’s estimates, we predicted the DBR probability distribution in the screened Flemish honey bee populations. As such, this research paper reveals prospects of marker-assisted selection for *Varroa* drone brood resistance in honey bees.

## Results

### Population-wide drone brood sampling, drone pupae phenotyping and genotyping

Out of 162 different drone brood samples from 43 Flemish beekeepers, a total of 842 drone pupae with an age between 15 and 19 days were singly infested and phenotyped according to the reproduction status of the included foundress mite in the capped brood cell. Six hundred and ten pupae with a single reproducing foundress mite were categorized as phenotype ‘1’ (= no DBR), whereas 232 pupae with a single non-reproducing foundress mite were categorized as phenotype ‘0’ (= DBR). All drones in the data set were descendants from the 162 different queens, with at most 28 and at least 1 drone(s) from the same queen in the reproducing phenotype (‘1’) group and at most 9 and at least 1 drone(s) from the same queen in the non-reproducing phenotype (‘0’) group (S1 Fig). All 842 drone pupae were genotyped for the eight genetic SNP variants associated with mite non-reproduction in drone brood (DBR) by qPCR genotyping assays with dual-labeled probes^54^.

### Genotype–phenotype association, modelling and probability calculation

#### Novel eight-variant mixed-effect modelling on population-wide data set (model M1)

**Figure 1:**
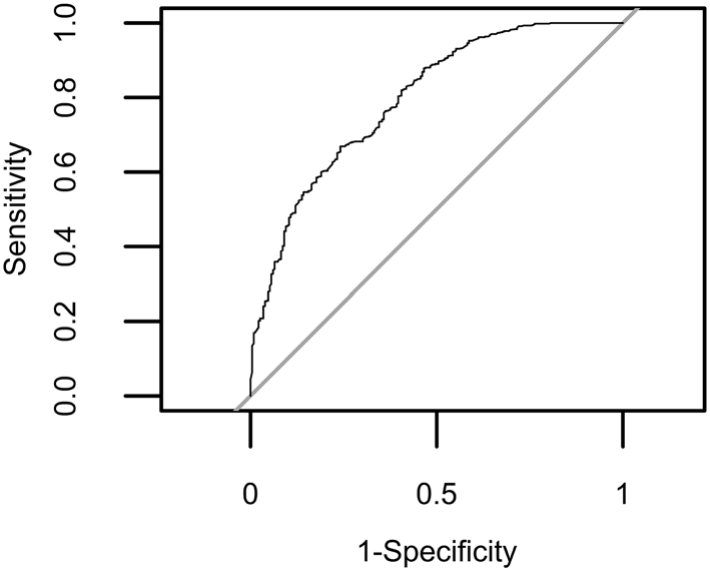
Evaluation of classification performance and determination of optimal cut-off by Receiver Operating Characteristic (ROC) curve analysis for the novel eight-variant logistic generalized linear mixed-effect model 1 (M1). ROC curve analysis resulted in optimal model cut-off at 1.198165.

The earlier published joint eight-variant model and its estimates was derived from only one hybrid colony from the Amsterdam Water Dunes in the Netherlands (DBBB colony)^66^. As such, this model could not directly be applied to our new population-overarching dataset containing offspring from another subspecies (*carnica ssp.*) and intra-colony and -beekeeper relationships between the sampled drones. Therefore, novel eight-variant mixed-effect modelling was performed on the obtained genotype–phenotype data set with all eight genotyped variants as fixed effects, the beekeeper as random effect and the reproduction status (non-reproduction = 0, reproduction = 1) of the included single foundress mite as outcome variable, and classification performance was tested by receiver operating characteristic (ROC) curve construction (Tables 1–2, Fig. 1).

**Table 1:** New eight-variant logistic generalized linear mixed-effect model 1 (M1).

SNP	LG	Position of variant on LG	Gene symbol	Gene name	Variant	Estim.	Std. error	Z	*p* value
1	LG1	26238027	LOC412088	Mucin-12 isoform X1	C > T	0.104	0.291	− 0.36	0.72
**2**	**LG1**	**26238077**	**LOC412088**	**Mucin-12 isoform X1**	**T > C**	**− 0.672**	**0.259**	**2.59**	0.01**
3	LG3	11110284	LOC724886	Uncharacterized protein LOC724886 isoform X2	G > A	0.312	0.199	− 1.57	0.12
**4**	**LG9**	**10054755**	**LOC100578770**	**Uncharacterized protein LOC100578770**	**T > C**	**0.440**	**0.196**	**− 2.24**	0.02*
5	LG9	10138359	LOC411744	Spectrin beta chain isoform X1	A > C	0.099	0.209	− 0.47	0.64
6	LG10	6310327	LOC408302	Solute carrier family 22 member 21	C > T	0.369	0.193	− 1.92	0.06
7	LG15	4736252	LOC410626	Sodium-coupled monocarboxylate transporter 1	C > T	− 0.495	0.400	1.24	0.22
**8**	**LG15**	**6143697**	**LOC551562**	**Dynein beta chain, ciliary**	**T > C**	**− 0.425**	**0.208**	**2.04**	0.04*

This model contains all eight previously described genetic variants as fixed effects (SNP1-8), the beekeeper as random effect and the reproduction status (0 or 1) of the included single foundress mite as outcome variable. The intercept of the model equaled 0.848. The ‘variant’ column describes the wild type (Wt) to variant type (Vt) allele conversion on DNA level. Fixed predictors that were significant at the 5% significance level are highlighted in bold. Significance codes: *p* ≤ 0.01**; *p* ≤ 0.05*. Linkage groups (LG), positions and gene symbols refer to reference genome Amel4.5. For locations of the tested variants on the Amel_HAv3.1 reference genome, see S1 Table. SNP numbers have been allocated in accordance with^54^.

**Table 2:** Contingency table of the true phenotype cases vs the predicted phenotype cases by the new eight-variant logistic generalized linear mixed-effect model 1 (M1).

Predictive model	Phenotypic group (truth)	
	1 (no DBR)	0 (DBR)
1	379	83
0	231	149

528 (379 + 149) out of 842 (63%) drones were correctly classified. Classification performance is based on a leave-one-out cross-validation (LOOCV) strategy.

#### Single variant tests for reduced three-variant model construction

Eight different single variant tests were performed with the single genetic variant as fixed effect (SNP1-8), the beekeeper as random effect and the reproduction status (non-reproduction = 0, reproduction = 1) of the included single foundress mite as outcome variable. These single variant tests were significant for SNP2, T > C (*p* ≤ 0.01**); SNP4, T > C (*p* ≤ 0.05*) and SNP6, C > T (*p* ≤ 0.05*) at the 5% significance level (Table 3).

**Table 3:** Single variant tests with an individual genetic variant as fixed effect (SNP1-8), the beekeeper as random effect and the reproduction status (0 or 1) of the included single foundress mite as outcome variable.

SNP	LG	Position of variant on LG	Gene symbol	Gene name	Variant	*p* value
1	LG1	26238027	LOC412088	Mucin-12 isoform X1	C > T	0.24
**2**	**LG1**	**26238077**	**LOC412088**	**Mucin-12 isoform X1**	**T > C**	0.01**
3	LG3	11110284	LOC724886	Uncharacterized protein LOC724886 isoform X2	G > A	0.13
**4**	**LG9**	**10054755**	**LOC100578770**	**Uncharacterized protein LOC100578770**	**T > C**	0.02*
5	LG9	10138359	LOC411744	Spectrin beta chain isoform X1	A > C	0.32
**6**	**LG10**	**6310327**	**LOC408302**	**Solute carrier family 22 member 21**	**C > T**	0.03*
7	LG15	4736252	LOC410626	Sodium-coupled monocarboxylate transporter 1	C > T	0.35
8	LG15	6143697	LOC551562	Dynein beta chain, ciliary	T > C	0.09

The ‘variant’ column describes the wild type (Wt) to variant type (Vt) allele conversion on DNA level. Single variant tests that were significant at the 5% significance level are highlighted in bold. Significance codes: *p* ≤ 0.01**; *p* ≤ 0.05*. Linkage groups (LG), positions and gene symbols refer to reference genome Amel4.5. For locations of the tested variants on the Amel_HAv3.1 reference genome, see S1 Table. SNP numbers have been allocated in accordance with^54^.

#### Logistic generalized linear mixed-effect modelling for reduced three-variant model construction on population-wide data set (model M2)

**Figure 2:**
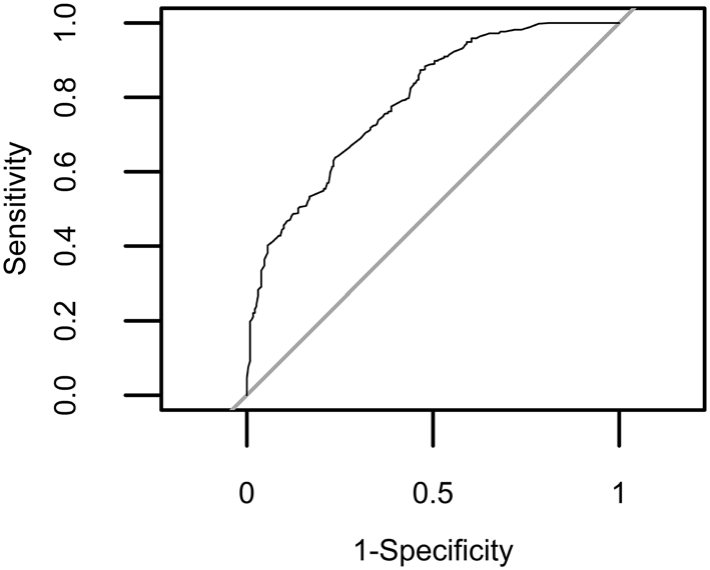
Evaluation of classification performance and determination of optimal cut-off by Receiver Operating Characteristic (ROC) curve analysis for the reduced three-variant model M2. ROC curve analysis resulted in optimal model cut-off at 0.630785.

In this study, two logistic generalized linear mixed-effect models have been constructed to test the classification performance of the genetic variants associated with *Varroa* non-reproduction in drone brood for the actual *Varroa* reproduction status. The previously described model M1 contained all eight genetic variants as fixed effects and the beekeeper as random effect (cfr. Table 1) and classified 63% of the phenotyped drones correctly (cfr. Table 2). Here, we describe a new reduced model, M2, containing only the three genetic variants that were significant in the single variant tests as fixed effects (i.e. SNP2, SNP4 and SNP6), the beekeeper as random effect and the reproduction status (non-reproduction = 0, reproduction = 1) of the included single foundress mite as outcome variable (Tables 4–5, Fig. 2).

**Table 4:** Reduced logistic generalized linear mixed-effect model 2 (M2).

SNP	LG	Position of variant on LG	Gene symbol	Gene name	Variant	Estim	Std.Error	Z	*p* value
2	LG1	26238077	LOC412088	Mucin-12 isoform X1	T > **C**	− 0.5599	0.1953	2.87	0.004**
4	LG9	10054755	LOC100578770	Uncharacterized protein LOC100578770	**T** > C	0.4581	0.1908	− 2.40	0.02*
6	LG10	6310327	LOC408302	Solute carrier family 22 member 21	**C** > T	0.3995	0.1903	− 2.10	0.04*

In this model, only the three genetic variants that were significant in the single variant tests were used as fixed effects (SNP2, SNP4 and SNP6), the beekeeper as random effect and the reproduction status (0 or 1) of the included single foundress mite as outcome variable. The ‘variant’ column describes the wild type (Wt) to variant type (Vt) allele conversion on DNA level. The intercept of the model equaled 0.8461. Favorable alleles are highlighted in bold. Significance codes: *p* ≤ 0.01**; *p* ≤ 0.05*. Linkage groups (LG), positions and gene symbols refer to reference genome Amel4.5. For locations of the tested variants on the Amel_HAv3.1 reference genome, see S1 Table. SNP numbers have been allocated in accordance with^54^.

**Table 5:** Contingency table of the true phenotype cases vs the predicted phenotype cases by the new reduced logistic generalized linear mixed-effect model 2 (M2).

Predictive model	Phenotypic group (truth)	
	1 (no DBR)	0 (DBR)
1	528	119
0	82	113

641 (528 + 113) out of 842 (76%) drones were correctly classified. Classification performance is based on a leave-one-out cross-validation (LOOCV) strategy.

There was no significant difference in classification performance between M1 and M2 at the 5% significance level (DeLong’s test for two correlated ROC curves; *p* > 0.05). The area under the ROC curve (AUC) for M1 was 0.7955059, while the AUC for M2 equaled 0.7909023 (cfr. Figs. 1 and 2, Table 6). The threshold, sensitivity and specificity of the reduced three-variant model (M2) was 0.630785, 0.487 and 0.866 respectively (Fig. 2, Table 6). This reduced logistic generalized linear model with three genetic variants as predictors classified 641 of the 842 phenotyped drones (76%) correctly (Tables 5 and 6).

Based on the reduced three-variant model M2 and its estimates, the probability on mite non-reproduction or DBR for each of the eight possible drone genotypes could be calculated (Table 7).

**Table 6:** Comparison of classification performance of model M1 and M2.

Model	Threshold	Specificity	Sensitivity	AUC	Prediction (%)
M1 (all 8 variants)	1.198165	0.621	0.642	0.7955059	63
M2 (reduced)	0.630785	0.866	0.487	0.7909023	76
DeLong’s test	*p* value = 0.43

**Table 7:** Probability on DBR for each of the eight possible drone genotypes according to the reduced three-variant model (M2).

Drone genotype ABC	P (DBR for drone genotype ABC)
Wt/Vt/Vt	0.154
Wt/Vt/Wt	0.213
Wt/Wt/Vt	0.223
Vt/Vt/Vt	0.242
Wt/Wt/Wt	0.300
Vt/Vt/Wt	0.322
Vt/Wt/Vt	0.335
Vt/Wt/Wt	0.429

Drone genotype ABC is represented in the format A/B/C with A the allele for SNP2; B the allele for SNP4 and C the allele for SNP6. *Wt =* wild type, *Vt* = variant type. Probabilities are sorted in ascending order, with the lowest DBR probability by genotype Wt/Vt/Vt (15.4%) and the highest DBR probability by genotype Vt/Wt/Wt (42.9%).

### Allelic frequency analysis by genotyping honey bee queens from Flanders

**Figure 3:**
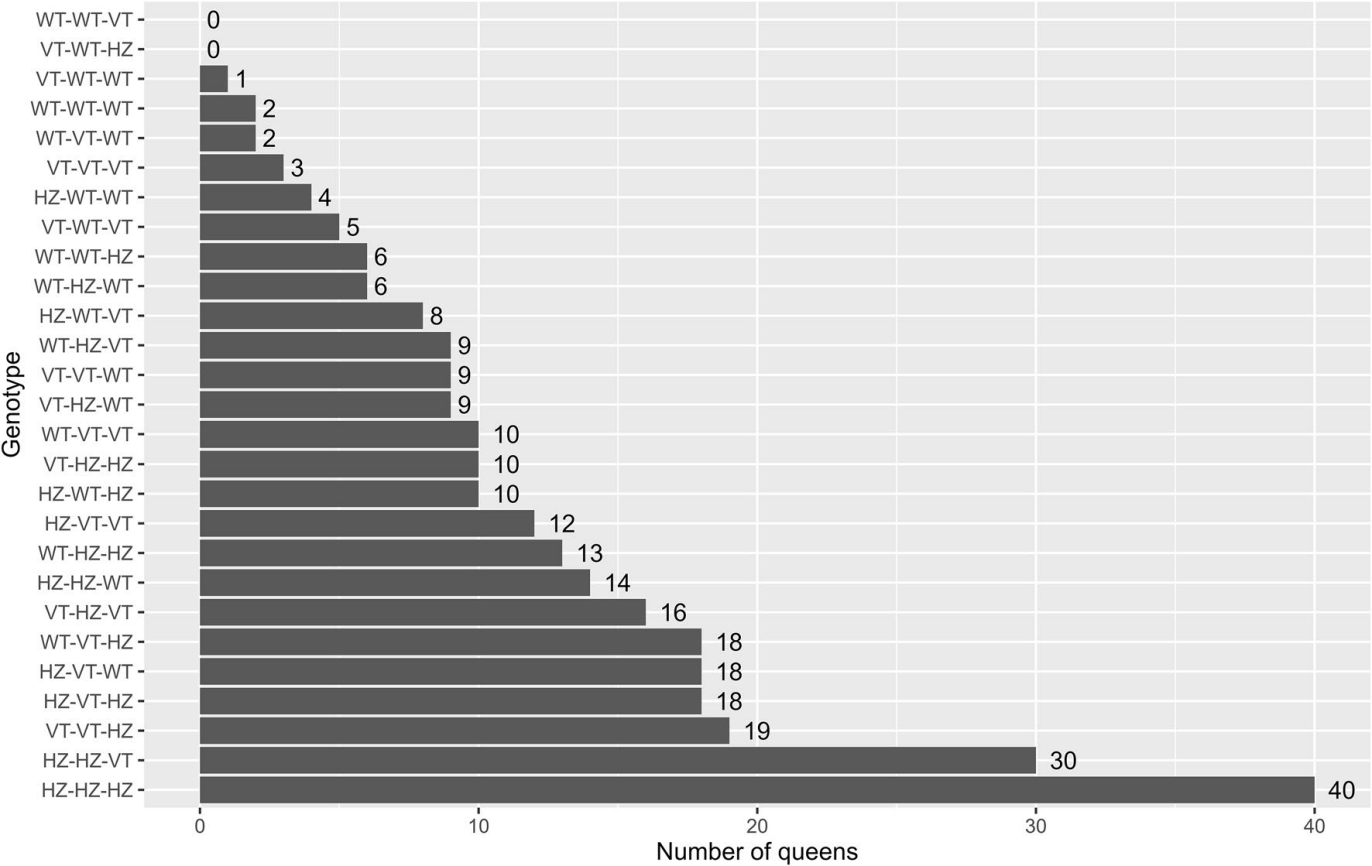
Absolute number of screened queens with each of the 27 possible genotypes for the three variants of reduced model M2 (N = 292). WT = homozygous wild type; VT = homozygous variant type; HZ = heterozygous. Queen genotypes are coded as X–Y–Z with X the homo-/heterozygosity for SNP2; Y the homo-/heterozygosity for SNP4 and Z the homo-/heterozygosity for SNP6. Most screened queens were heterozygous for the three genetic variants of model M2.

Over two consecutive years (2021–2022), 292 different honey bee queens from 54 Flemish beekeepers were genotyped for the three genetic variants of reduced model M2 through gDNA extraction from 30 pooled drone hind legs and qPCR genotyping assays with dual-labeled probes (Tables S3 and 8, Fig. 3). Table 8 shows the individual distribution of genotypes and allelic frequencies of the variant-type alleles for the three genetic variants of M2 in the screened queens. Figure 3 shows the absolute number of queens with each of the 27 possible genotypes (N = 292).

**Table 8:** Distribution of genotypes and allelic frequencies of the variant-type alleles for the three genetic variants from model M2 in the 292 screened queens from Flanders.

SNP	LG	Position of variant on LG	Gene symbol	Gene name	Genotype			Allelic freq. Vt
					Wt/Wt	Wt/Vt	Vt/Vt	
2	LG1	26238077	LOC412088	Mucin-12 isoform X1	66	154	72	0.51
4	LG9	10054755	LOC100578770	Uncharacterized protein LOC100578770	36	147	109	0.63
6	LG10	6310327	LOC408302	Solute carrier family 22 member 21	65	134	93	0.55

*Wt* = wild type, *Vt =* variant type. Note that the Vt allele is unfavorable for SNP4 and SNP6 (cfr. Table 4). The allele frequencies of the favorable alleles are thus 0.51, 0.37 and 0.45 for SNP2; SNP4 and SNP6 respectively. Linkage groups (LG), positions and gene symbols refer to reference genome Amel4.5. For locations of the tested variants on the Amel_HAv3.1 reference genome, see S1 Table. SNP numbers have been allocated in accordance with^54^.

### Predicted colony-level DBR probabilities in drone brood from honey bee queens from Flanders

**Figure 4:**
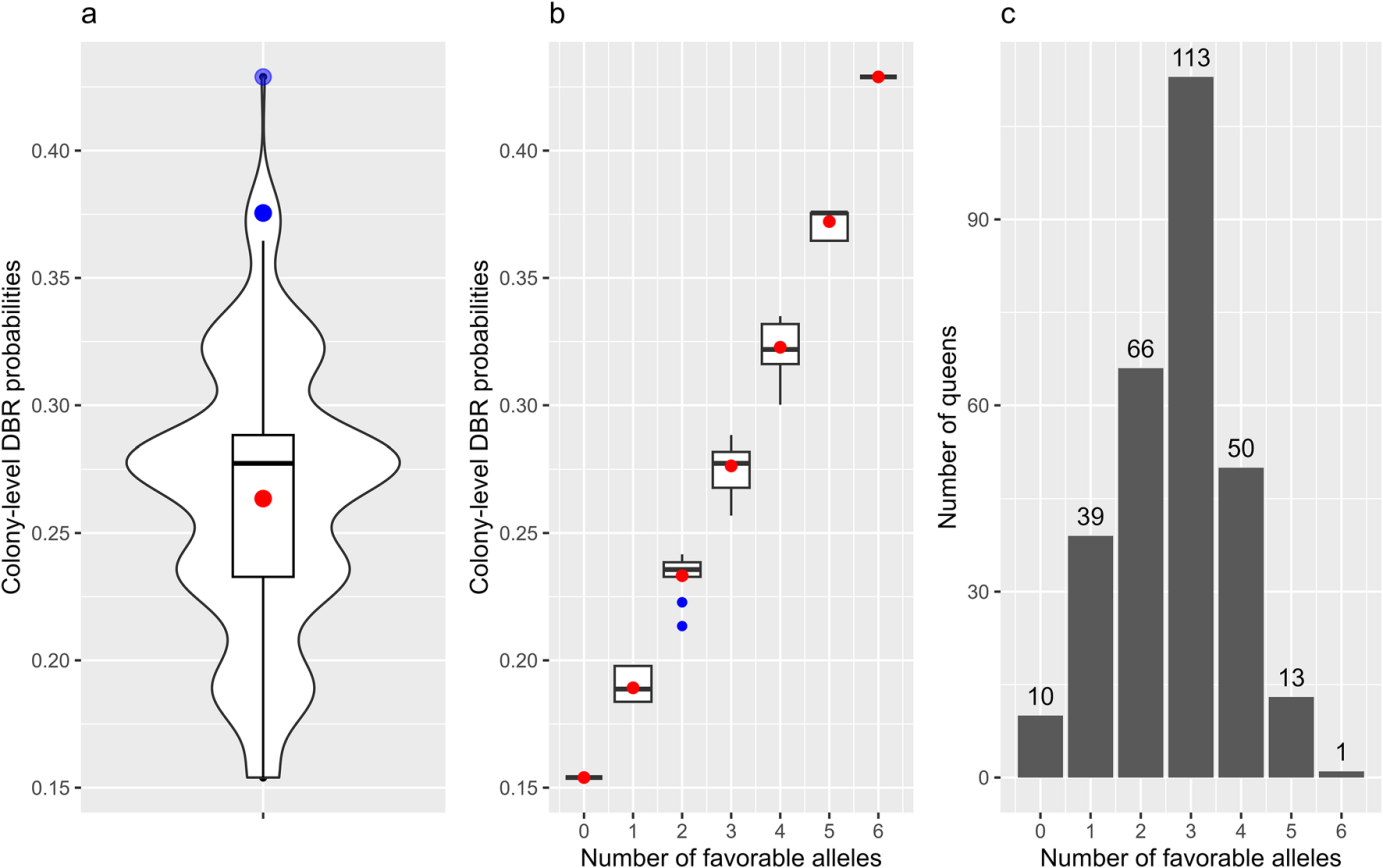
(**a**) Distribution of predicted colony-level DBR probabilities over all 292 genotyped queens from Flanders, (**b**) distributions of colony-level DBR probabilities in function of the number of favorable alleles in the queen and (**c**) absolute number of queens in function of the number of favorable alleles. (**a**) The mean and median colony-level DBR probability over all 292 screened queens was 0.26 and 0.28 respectively. (**b**) A queen with N favorable alleles will always have a lower colony-level DBR probability than a queen with N + 1 favorable alleles, independent of which genetic variants are favorable and which are not. Means are indicated in red, outliers in blue. (**c**) Absolute number of screened queens in function of the number of favorable alleles (N = 292). Only one queen was homozygous favorable for all three genetic variants of model M2.

For each of the 292 genotyped honey bee queens, we calculated the probability on mite non-reproduction in the respective colony’s total drone brood using reduced model M2 (Fig. 4a). The mean and median predicted colony-level DBR probability in this screening was 0.26 and 0.28 respectively. Ranking the queens only based on the number of favorable alleles (out of six alleles) ranks them for predicted colony-level DBR probabilities as well (Fig. 4b). For instance, if we want to select only colonies that have colony-level DBR probabilities higher than or equal to 0.3, we may simply select all queens with four or more favorable alleles, independent of which genetic variants are favorable and which are not. In this screening, a total of 64 queens complied with this condition (Fig. 4c).

## Discussion

As previously mentioned, the original joint eight-variant model published by^66^ was derived from only one hybrid colony from the Amsterdam Water Dunes in The Netherlands. Of the four F1 hybrid *Varroa*-resistant/*Varroa*-sensitive queens generated, only the queen derived from the Amsterdam Water Dune stock produced drone brood offspring in which the percentage of DBR differed significantly from a *Varroa*-sensitive control queen (that is 51 vs. 19% resp.)^66^. Sixty-four phenotyped drones from this hybrid queen were subsequently used for comparative WES analysis and variant model construction by elastic-net penalized regression. Thus, contrary to our new colony-wide data set, the drones in this preceding study were all descendants from a single honey bee queen, skipping representation of ancestrally diverse population offspring and divergent genetic background in the data set for genotype–phenotype association analysis. Therefore, it was unknown whether the reported eight-variant model remained valid on a population-wide scale and a different subspecies or, with other words, was colony-specific. As such, this new study aimed for the validation of the previously discovered eight-variant model on an independently obtained, representative and population-encompassing genotype–phenotype data set. By novel eight-variant modelling with random effects to account for relatedness of samples in the population-wide data set, we found that only three of the eight previously discovered variants were significant predictors in the new eight-variant model (i.e. SNP2, SNP4 and SNP8). Moreover, the sign of the estimated effect size for SNP2 and SNP8 switched from positive in the previously published joint eight-variant model to negative in the new model, while the one for SNP4 stayed positive in both. Consequently, SNP2 and SNP8 should now be considered as protective mutations according to the new eight-variant model, while SNP4 remains a risk mutation. SNP6 was almost significant as predictor in the novel eight-variant model (*p* = 0.06), and showed up as a significant fixed effect in the single variant tests (*p* value < 0.05*) and reduced three-variant model (*p* value < 0.05*). The sign of the estimate of SNP6 switched from negative in the original eight-variant model to positive in the reduced three-variant model, changing it from a protective variant to a risk mutation.

The concept of ‘genetic background’ in an organism is defined as the set of so-called ‘modifier genes’ and their genotypes that interact with the gene or variation of interest, influencing the phenotype of interest too. Background effects manifest when genetically different individuals exhibit varied phenotypic outcomes despite comprising the same genotype for certain residing or induced mutation(s), which could be explained by the epistasis effect^70–73^. This latter effect refers to the phenomenon in which the impact of a gene mutation relies on the genotype of one or more other modifier genes interacting with the considered gene. In mice for instance, mutations induced on different ancestral backgrounds or strains have already resulted in several confounding outcomes. One example is that of the diabetes mutation Lepr^db^, that results in obesity with transient diabetes when introduced in a C57BL/6J strain, but obesity with overt diabetes when introduced in a C57BLKS/J strain^74^. Regarding our findings, it is not inconceivable that genetic background may underlie the sign switches of the estimates between the previously published model and the new models described in the current study. More specifically, the originally published joint eight-variant model was derived from a single colony arising from a Darwinian Black Bee Box (DBBB) selection program, while the Flemish population-wide sampling strategy applied in the current study included diverse genetic backgrounds from multiple *A. mellifera carnica* colonies^66,67,75^. DBBB selection involves leveraging natural selection in honey bees, in this case the European dark bee or *Apis mellifera mellifera*, to enhance resistance by discontinuing mite treatment in managed colonies stationed on isolated locations^67^. Its core principles revolve around mating within the population, specifically between the colonies' own virgin queens and drones, and selecting colonies based on their survival and prolific development^67^. As such, the genetic background of the Amsterdam Water Dunes population and the therefrom derived hybrid queen used for the original eight-variant model construction may be considered very niche and different from these in our new population-wide sample set.

As the new models described in the current study are derived from and thus, up to now, are only applicable on Carniolan bees, we should note that similar population-wide genotype–phenotype association studies should be performed for other subspecies in Europe (such as *Apis mellifera mellifera*, *Apis mellifera ligustica* and *Apis mellifera iberiensis*) to verify the predictive value of the eight variants for the DBR trait^76,77^*.* However, due to the evolutionary relatedness between *Apis mellifera carnica* and *Apis mellifera ligustica* (C lineage), we expect similar results for the Italian subspecies as described in this study for Carniolan bees^77^. Although *Apis mellifera iberiensis* was morphologically and predominantly categorized in the M lineage together with *Apis mellifera mellifera* (European dark bee), other studies showed that the Spanish genotype rather finds its evolutionary origin in the African A lineage, especially when considering bees from the south-western part of the Iberian Peninsula^76–80^. Thus, both subspecies should be handled separately in future equivalent studies.

No significant difference in classification performance between the new eight-variant model M1 and reduced three-variant model M2 could be found at the 5% significance level, although M2 did classify 13% more drones correctly. In addition, when focusing on the DBR phenotype only, i.e. phenotype “0”, the positive predictive value (PPV) or fraction of true cases among the predicted cases of M2 was much higher than the PPV of M1 (58 vs. 39% respectively). Given this, together with the fact that screening and selecting for three genetic variants is practically more feasible than eight, future MAS-programs can benefit from the reduced three-variant model reported in this study. Using the reduced three-variant model’s estimations and distribution of the 292 Flemish queen genotypes, we predicted an average and median colony-level DBR probability over all screened colonies of 0.26 and 0.28 respectively (Fig. 4). Due to very low drone brood sample infestation levels, accurate DBR phenotypic data on colony level is lacking to compare the predicted colony-level DBR probabilities of the screened queens with their actual DBR scores^48,52^. Although colony-level DBR calculations are independent from *Varroa* infestation levels, a sufficient amount of single infested cells (SICs) in the brood samples are required for accurate and precise estimation of this phenotype^48,52^. However, the range of our predicted colony-level DBR probabilities in drone brood, i.e. from 15.4% up to 42.9%, is comparable to those reported by phenotyping in other studies^49,81–84^.

Due to the haplodiploid system in honey bees, colonies can be screened for supply of paternal lines by pooling the respective queens’ drones’ hind legs and determining the homo-or heterozygosity of each queen for the three genetic variants in model M2, as performed in this study (Table 8, Fig. 3). Input of beneficial alleles via the patriline guarantees input of beneficial alleles in the F1 fertilized eggs, which is beneficial for queen rearing, and also guarantees beneficial alleles in that F1 queen’s drones (F2). Our results show that thirty pooled drone legs are sufficient for accurate queen SNP-genotyping by qPCR’s with dual-labelled probes, which is similar to the sample size used in another study using a 100K SNP chip for queen genotyping by pooling drones^85^. When pooling only ten legs from drones descending from a heterozygous queen, the probability of proportions of wild-type and variant-type drones being in the interval of [30–70%] is 0.891. With 30 pooled legs, this probability increases up to 0.984. Lab tests with post-genotyped pooled individuals showed that proportions of wild-type and variant-type drones in the interval of [30–70%] are easily capable of distinguishing heterozygous queens from homozygous queens for a specific variant of interest when using the qPCR’s with dual-labeled probes (S2 Fig). Moreover, the probability of sampling 30 drones with the same allele from a heterozygous queen is about 9.3^–10^ (0.5^30^), while this probability is 0.00098 (0.5^10^) when sampling only ten drones. Instead of pooling drones, non-destructive queen genotyping can also be performed through gDNA extraction from the queen’s feces, exuviae or queen cell, which can be performed immediately after queen emergence^85,86^. On the other hand, genetically screening colonies for queen rearing can be fulfilled by pooling the queens’ worker bees’ hind legs and determining the percentages of beneficial alleles of the three genetic variants in the gDNA pool by running intraplate calibration curves with known percentages of alleles. Colonies with high percentages of beneficial alleles in their worker gDNA pools highlight high probabilities on beneficial alleles in queens reared therefrom. Once paternal and maternal lines are selected, mating may be controlled by means of artificial insemination or isolated mating stations. However, the speed of allele fixation in honey bee populations being framed in an organized selection program is difficult to predict and simulate, as this highly depends on many other factors, such as the consistency of the starting population, number of participating colonies in the breeding program, undesired allele input and other traits of interest. It is hard to predict to what extent marker-assisted selection (MAS) based on the three genetic variants reported here will contribute to overall holistic colony resistance, when ‘holistic colony resistance’ is being defined as the combination of all *Varroa*-resistance traits (e.g. MNR, VSH, grooming behavior, etc.). In this case, it is important to thoroughly consider and ‘weight’ the colony’s performance on these traits, as well as the presence of the beneficial alleles reported here.

The main advantages of the qPCR with dual-labeled probes and the above proposed protocols for selection of paternal and maternal lines are their accessibility, ease of use and implementation, rapidity and cost. Although comparative Genome-Wide Association Studies (GWAS) or high-density SNP chip analyses between colony-encompassing drone pupae expressing the DBR trait and non-DBR drone pupae could potentially reveal more and/or other significant phenotype-associated markers, the application of Whole Genome Sequencing (WGS) or SNP chips for high rate routine screening in honey bee sciences is less accessible, more complicated, more expensive and more time-consuming compared to qPCR’s with dual-labeled probes, especially for small research institutes^87^. For example, the 100K High-Density Honey Bee (HDHB) SNP chip from Jones et al. contains over 100K honey bee SNPs, including 498 SNPs associated with *Varroa*-sensitive hygiene and 8478 SNPs associated with *Varroa* resistance^85^. Population-wide association studies similar to the one reported here could be performed using this SNP chip and reveal more SNP’s associated with the trait of interest, although it should be noted that the eight DBR-associated SNP’s targeted in the current study resulted from an elastic-net penalized regression on more than 140.000 SNP’s that were not significant on genome-wide scale in the previous comparative WES study^66^. Same issues could be encountered when performing comparative SNP chip—or GWAS analyses, demanding for a joint variant modelling as well, which will considerably reduce the number of phenotype-associated variants too.

The use of genetic markers in MAS has emerged as a powerful tool in countless selective breeding programs. By identifying specific regions or variants in the honey bee genome associated with desirable traits, such as *Varroa* resistance, genetic markers enable breeders to make efficient targeted selections and allow for a high-throughput bottom-up selective breeding approach. It is important to note that the successful implementation of genetic markers in MAS relies on a comprehensive understanding of the genetic basis of the targeted trait. In the case of the three genetic variants associated with DBR in Flanders reported in this study, further research should clarify the role of the genetic markers in Worker Brood Resistance (WBR, i.e. MNR in worker brood), but also the molecular basis of the marker-associated genes resulting in or influencing the DBR phenotype.

## Materials and methods

### Phenotyping for genotype–phenotype association study

Over a time period of 2 years (2020 and 2021), 162 different *A. mellifera* colonies from 43 Flemish *carnica* ssp. beekeepers were sampled for at least two square decimeter of capped drone brood with an age between 15 and 19 days (S2 Table). Each brood sample was frozen immediately after collection by the beekeeper and kept in a cold chain (− 20 °C) for transport to the laboratory. During brood analysis, capped cells were opened under a microscope and inspected for the presence of foundress mites, male mites and/or daughter mites. Pupae with a single reproducing foundress mite (i.e. at least one offspring mite) were categorized as phenotype ‘1’ (= no DBR), whereas pupae with a single non-reproducing foundress mite (i.e. total absence of offspring) were categorized as phenotype ‘0’ (= DBR). Per brood sample, analysis was ceased when 200 cells were dissected. All sampled colonies were managed according to standard beekeeping practices.

### Single thorax gDNA extractions

From each of the 842 phenotyped drone pupae, the thorax was dissected and individually homogenized with 0.5 mL lysis buffer (100 mM NaCl; 20 mM Tris–HCl, pH 8; 25 mM EDTA, pH 8; 0.5% SDS), metal- and zirconium beads during 1 min at 30 Hz with a PowerLyzer® 24 Homogenizer. After incubation with 10 µL proteinase K (20 mg/mL) at 56 °C for 4 h, gDNA was extracted by addition of an equal volume of phenol:chloroform:isoamylalcohol and centrifugation at 12000g for 30′ at 4 °C, followed by transfer and extraction of the supernatant with an equal volume of chloroform and centrifugation at 12000g for 15′ at 4 °C. The gDNA in the transferred supernatant was precipitated by addition of two volumes of ice cold 100% ethanol and overnight incubation at − 20 °C. After centrifugation at 12000 g for 30′, the DNA pellet was washed with 70% ethanol, air-dried and resuspended in 100 µL DNase/RNase free water.

### qPCR assays with dual-labeled probes for variant genotyping

The qPCR assays with dual-labeled probes of Bouuaert et al. (2021) were used for genotyping^54^. Briefly, for each gDNA sample, genotyping assays were performed in a total volume of 10 µL with 1 × KEY buffer, 250 nM of each primer, 250 nM of each dual-labeled probe, 200 µM of each dNTP, 0.5 U TEMPase Hot Start DNA Polymerase (VWR) and 20 ng gDNA. Primer and probe sequences can be found in^54^. The Bio-Rad C1000™ Thermal Cycler with CFX96™ Real-Time System was set at one cycle of 95 °C for 14′40″, followed by 60 cycles of [95 °C for 20″ followed by 40″ of the assay-specific annealing/elongation/signal detection temperature]^54^. Data analysis and allelic discrimination plot construction was done with the Bio-Rad CFX Manager 3.1 Software.

### Genotype–phenotype association and modelling

RStudio version 4.2.2 was used for data exploration and—visualization. All statistical tests were checked for and complied with the required assumptions. For modelling, analyses were conducted in R version 4.1.3 (“One Push-Up”), with the packages “multcomp”, “cutpointr”, “lme4”, “lmerTest” and “pROC”. For the single variant tests, a logistic mixed-effect model with the reproduction status of the included single foundress mite (non-reproduction (DBR) = 0, reproduction (no DBR) = 1) as binary outcome variable, the beekeeper as random effect and a single variant as predictor was compared to a logistic mixed-effect model with the reproduction status of the foundress mite as outcome variable and the beekeeper as random effect by means of ANOVA testing. When significant, a post hoc test was used with a Tukey correction for multiple testing. To evaluate the classification performance of the variants for *Varroa* reproduction, the prediction of a logistic generalized linear mixed-effect model containing (1) all eight genetic variants as fixed effects and the beekeeper as random effect and (2) the subset of significant genetic variants from the single variant tests as fixed effects and the beekeeper as random effect was compared with the real reproduction status using a leave-one-out cross validation strategy (LOOCV). Optimal cutoff points for classification were based on the Youden index. Classification performance was analyzed using Delong’s test. None of the constructed models did converge with ‘colony’ as random variable (due to too few drones per colony). As colonies from the same beekeeper are genetically more related, beekeeper was used as a simple approximation. Throughout all analyses, significance was set at α ≤ 0.05.

### DBR probability calculations based on reduced three-variant model M2

Based on the reduced three-variant model M2 and its estimates (cfr. Table 4), the probability on mite non-reproduction or DBR for each of the eight possible drone genotypes could be calculated with formulas (1) and (2):

1 $${\text{P}}\left( {{\text{DBR}}\;{\text{for}}\;{\text{drone}}\;{\text{genotype}}\;{\text{ABC}}} \right) = 1 - \left( {\frac{{\exp \left( {\text{Y}} \right)}}{{1 + \exp \left( {\text{Y}} \right)}}} \right)$$

2 $$\begin{gathered} {\text{with Y}} = 0.8461 + {\text{A*}}\left( { - 0.5599} \right) + {\text{B*}}0.4581 + {\text{C*}}0.3995 \hfill \\ \quad \quad \quad \quad \quad \quad \quad \quad \quad \quad \quad \quad \quad \quad {\text{A}},{\text{B}},{\text{C}} = 0\;{\text{if}}\;{\text{Wt}}\;{\text{for}}\;{\text{SNP}}2;\;{\text{SNP}}4\;{\text{and}}\;{\text{SNP}}6\;{\text{resp}}. \hfill \\ \quad \quad \quad \quad \quad \quad \quad \quad \quad \quad \quad \quad \quad \quad {\text{A}},{\text{B}},{\text{C}} = 1\;{\text{if}}\;{\text{Vt}}\;{\text{for}}\;{\text{SNP}}2;\;{\text{SNP}}4\;{\text{and}}\;{\text{SNP}}6\;{\text{resp}}. \hfill \\ \end{gathered}$$

### Pooled drone leg gDNA extractions for queen genotyping and allelic frequency analysis

For each of the 292 bee queens screened in the allelic frequency analysis, 30 hind legs from 30 different drone pupae were pooled in an Eppendorf tube containing 180 µL ATL buffer from the QIAamp® DNA Micro Kit (Qiagen). After overnight incubation at 56 °C with 20 µL proteinase K, gDNA was extracted according to the manufacturer’s instructions. gDNA was eluted in 50 µL DNase/RNase free water. Queen genotyping for SNP2; SNP4 and SNP6 was performed as previously described by qPCR assays with dual labelled probes using the pooled drone gDNA as template. For each variant, allelic discrimination plots were constructed by plotting the end-point Relative Fluorescence Units (end-RFU) values of FAM (fluorescein; wild-type fluorophore) against the end-RFU values of TR (Texas Red; variant-type fluorophore) for all pooled drone leg samples. Based on these allelic discrimination plots, the genotypes of the respective queens were reconstructed. For each of the three variants, the frequency of the variant type allele was calculated as (number of heterozygous queens + 2*number of homozygous variant type queens)/(2*total number of screened queens).

### Predicted colony-level DBR probabilities in drone brood from honey bee queens from Flanders

Based on the genotype of a queen, the probability on mite non-reproduction in that queen’s offspring drone brood could be predicted by using model M2. First, the probability on each of the eight possible drone genotypes was calculated for each queen. Next, these probabilities on the different drone genotypes were multiplied with the corresponding probabilities on mite-non reproduction for the respective drone genotypes. The probability on mite non-reproduction in the colony is the sum of these multiplied probabilities:

$${\text{P}}\left( {{\text{colony}} - {\text{level}}\;{\text{DBR}}} \right) = \sum \left( {{\text{P}}\left( {{\text{drone}}\;{\text{genotype}}\;{\text{ABC}}} \right){\text{*P}}\left( {{\text{DBR}}\;{\text{for}}\;{\text{drone}}\;{\text{genotype}}\;{\text{ABC}}} \right)} \right)$$

### Supplementary Information

Supplementary Information 1.

Supplementary Information 2.

Supplementary Information 3.

Supplementary Information 4.

Supplementary Information 5.

## Data Availability

The datasets generated and/or analyzed during the current study are available from the corresponding author on reasonable request.

## References

[1] Oldroyd BP (1999). Coevolution while you wait: *Varroa jacobsoni*, a new parasite of western honeybees. Trends Ecol. Evol..

[2] Rosenkranz P, Aumeier P, Ziegelmann B (2010). Biology and control of Varroa destructor. J. Invertebr. Pathol..

[3] Le Conte Y, Ellis M, Ritter W (2010). Varroa mites and honey bee health: Can Varroa explain part of the colony losses?. Apidologie.

[4] Genersch E (2010). The German bee monitoring project: A long term study to understand periodically high winter losses of honey bee colonies. Apidologie.

[5] Dahle B (2010). The role of *Varroa destructor* for honey bee colony losses in Norway. J. Apic. Res..

[6] Carreck NL, Bell BV, Martin SJ (2010). Honey bee colony collapse and changes in viral prevalence associated with *Varroa destructor*. J. Apic. Res..

[7] De la Rua P, Jaffe R, Dall'Olio R, Munoz I, Serrano J (2009). Biodiversity, conservation and current threats to European honeybees. Apidologie.

[8] Trouiller J, Arnold G, Chappe B, Le Conte Y, Masson C (1992). Semiochemical basis of infestation of honey-bee brood by *Varroa jacobsoni*. J. Chem. Ecol..

[9] Trouiller J, Arnold G, Le Conte Y, Masson C, Chappe B (1991). Temporal pheromonal and kairomonal secretion in the brood of honeybees. Naturwissenschaften.

[10] Rickli M, Diehl PA, Guerin PM (1994). Cuticle alkanes of honeybee larvae mediate arrestment of bee parasite *Varroa jacobsoni*. J. Chem. Ecol..

[11] Le Conte Y (1989). Attraction of the parasitic mite Varroa to the drone larvae of honey bees by simple aliphatic esters. Science.

[12] Liu JM (2023). The Role of honey bee derived aliphatic esters in the host-finding behavior of *Varroa destructor*. Insects.

[13] Fuchs S (1990). Preference for drone brood cells by *Varroa jacobsoni* oud in colonies of *Apis mellifera* carnica. Apidologie.

[14] Boot WJ, Schoenmaker J, Calis JNM, Beetsma J (1995). Invasion of *Varroa jacobsoni* into drone brood cells of the honey-bee *Apis mellifera*. Apidologie.

[15] Aumeier P, Rosenkranz P, Francke W (2002). Cuticular volatiles, attractivity of worker larvae and invasion of brood cells by Varroa mites. A comparison of Africanized and European honey bees. Chemoecology.

[16] Boot WJ, Driessen RG, Calis JNM, Beetsma J (1995). Further observations on the correlation between attractiveness of honey-bee brood cells to *Varroa jacobsoni* and the distance from larva to cell rim. Entomol. Exp. Appl..

[17] Message D, Goncalves LS (1995). Effect of the size of worker brood cells of Africanized honey-bees on infestation and reproduction of the ectoparasitic mite *Varroa jacobsoni* Oud. Apidologie.

[18] Oddie MAY, Neumann P, Dahle B (2019). Cell size and *Varroa destructor* mite infestations in susceptible and naturally-surviving honeybee (*Apis mellifera*) colonies. Apidologie.

[19] Le Conte Y, Arnold G (1988). Etude du thermopréférendum de *Varroa jacobsoni* Oud. Apidologie.

[20] Ifantidis MD (1983). Ontogenesis of the mite *Varroa jacobsoni* in worker and drone honeybee brood cells. J. Apic. Res..

[21] Martin SJ (1994). Ontogenesis of the mite *Varroa jacobsoni* Oud. in worker brood of the honeybee *Apis mellifera* L. under natural conditions. Exp. Appl. Acarol..

[22] Martin SJ (1995). Ontogenesis of the mite *Varroa jacobsoni* Oud. in drone brood of the honeybee *Apis mellifera* L. under natural conditions. Exp. Appl. Acarol..

[23] Calderon RA, Zamora LG, Van Veen JW, Quesada MV (2007). A comparison of the reproductive ability of *Varroa destructor* (Mesostigmata: Varroidae) in worker and drone brood of Africanized honey bees (*Apis mellifera*). Exp. Appl. Acarol..

[24] Calderon RA, Urena S, van Veen JW (2012). Reproduction of *Varroa destructor* and offspring mortality in worker and drone brood cells of Africanized honey bees. Exp. Appl. Acarol..

[25] Ghamdi AA (2003). Reproductive biology of *Varroa jacobsoni* Oud. in worker and drone brood of the honey bee *Apis mellifera* L. under Michigan conditions. Pak. J. Biol. Sci..

[26] Mondragon L, Martin S, Vandame R (2006). Mortality of mite offspring: a major component of *Varroa destructor* resistance in a population of Africanized bees. Apidologie.

[27] Martin SJ (1997). Life and Death of Varroa.

[28] Kurze C, Routtu J, Moritz RFA (2016). Parasite resistance and tolerance in honeybees at the individual and social level. Zoology.

[29] Mondet F (2020). Honey bee survival mechanisms against the parasite *Varroa destructor*: A systematic review of phenotypic and genomic research efforts. Int. J. Parasitol..

[30] Locke B (2016). Natural Varroa mite-surviving *Apis mellifera* honeybee populations. Apidologie.

[31] Rosenkranz P, Engels W (1994). Infertility of *Varroa jacobsoni* females after invasion into *Apis mellifera* worker brood as a tolerance factor against varroatosis. Apidologie.

[32] Rosenkranz P (1999). Honey bee (*Apis mellifera* L.) tolerance to *Varroa jacobsoni* Oud, South America. Apidologie.

[33] Locke B, Fries I (2011). Characteristics of honey bee colonies (*Apis mellifera*) in Sweden surviving *Varroa destructor* infestation. Apidologie.

[34] Fries I, Imdorf A, Rosenkranz P (2006). Survival of mite infested (Varroa destructor) honey bee (*Apis mellifera*) colonies in a Nordic climate. Apidologie.

[35] Locke B, Le Conte Y, Crauser D, Fries I (2012). Host adaptations reduce the reproductive success of *Varroa destructor* in two distinct European honey bee populations. Ecol. Evol..

[36] Le Conte Y (2007). Honey bee colonies that have survived *Varroa destructor*. Apidologie.

[37] Oddie MAY, Dahle B, Neumann P (2017). Norwegian honey bees surviving *Varroa destructor* mite infestations by means of natural selection. PeerJ.

[38] Moritz RFA, Hanel H (1984). Restricted development of the parasitic mite *Varroa jacobsoni* Oud. in the cape honeybee *Apis mellifera* capensis Esch. Zeitschrift Fur Angewandte Entomologie-Journal of Applied Entomology.

[39] Strauss U, Dietemann V, Human H, Crewe RM, Pirk CWW (2016). Resistance rather than tolerance explains survival of savannah honeybees (*Apis mellifera scutellata*) to infestation by the parasitic mite *Varroa destructor*. Parasitology.

[40] Harbo JR, Hoopingarner RA (1997). Honey bees (Hymenoptera:Apidae) in the United States that express resistance to *Varroa jacobsoni* (Mesostigmata:Varroidae). J. Econ. Entomol..

[41] Harbo JR, Harris JW (2002). Suppressing mite reproduction: SMR an update. Bee Cult..

[42] Fuchs S (1994). Non-reproducing *Varroa jacobsoni* Oud. in honey bee worker cells—status of mites or effect of brood cells?. Exp. Appl. Acarol..

[43] Martin G (2023). Heritability of *Apis mellifera* recapping behavior and suppressed mite reproduction as resistance traits towards *Varroa destructor*. Front. Insect Sci..

[44] Harbo JR, Harris JW (1999). Heritability in honey bees (Hymenoptera : Apidae) of characteristics associated with resistance to *Varroa jacobsoni* (Mesostigmata : Varroidae). J. Econ. Entomol..

[45] Frey E, Odemer R, Blum T, Rosenkranz P (2013). Activation and interruption of the reproduction of *Varroa destructor* is triggered by host signals (*Apis mellifera*). J. Invertebr. Pathol..

[46] Garrido C, Rosenkranz P (2004). Volatiles of the honey bee larva initiate oogenesis in the parasitic mite *Varroa destructor*. Chemoecology.

[47] Garrido C, Rosenkranz P (2003). The reproductive program of female *Varroa destructor* mites is triggered by its host, *Apis mellifera*. Exp. Appl. Acarol..

[48] Mondet F (2020). Evaluation of suppressed mite reproduction (SMR) reveals potential for varroa resistance in european honey bees (*Apis mellifera* L.). Insects.

[49] von Virag A, Guichard M, Neuditschko M, Dietemann V, Dainat B (2022). Decreased mite reproduction to select *Varroa destructor* (Acari: Varroidae) resistant honey bees (Hymenoptera: Apidae): Limitations and potential methodological improvements. J. Econ. Entomol..

[50] Kovacic, M. Screening for low Varroa mite reproduction (SMR) and recapping in European honey bees (2017).

[51] Dietemann V (2013). Standard methods for Varroa research. J. Apic. Res..

[52] Eynard SE (2020). Descriptive analysis of the varroa non-reproduction trait in honey bee colonies and association with other traits related to varroa resistance. Insects.

[53] Dekkers JCM, Hospital F (2002). The use of molecular genetics in the improvement of agricultural populations. Nat. Rev. Genet..

[54] Bouuaert DC (2021). qPCR assays with dual-labeled probes for genotyping honey bee variants associated with Varroa resistance. BMC Vet. Res..

[55] Oxley PR, Spivak M, Oldroyd BP (2010). Six quantitative trait loci influence task thresholds for hygienic behaviour in honeybees (*Apis mellifera*). Mol. Ecol..

[56] Tsuruda JM, Harris JW, Bourgeois L, Danka RG, Hunt GJ (2012). High-resolution linkage analyses to identify genes that influence Varroa sensitive hygiene behavior in honey bees. Plos One.

[57] Spotter A, Gupta P, Nurnberg G, Reinsch N, Bienefeld K (2012). Development of a 44K SNP assay focussing on the analysis of a varroa-specific defence behaviour in honey bees (*Apis mellifera carnica*). Mol. Ecol. Resour..

[58] Spotter A, Gupta P, Mayer M, Reinsch N, Bienefeld K (2016). Genome-wide association study of a Varroa-specific defense behavior in honeybees (*Apis mellifera*). J. Hered..

[59] Harpur BA (2019). Integrative genomics reveals the genetics and evolution of the honey bee’s social immune system. Genome Biol. Evol..

[60] Sainsbury J (2022). Marker assisted selection for *Varroa destructor* resistance in New Zealand honey bees. Plos One.

[61] Arechavaleta-Velasco ME, Alcala-Escamilla K, Robles-Rios C, Tsuruda JM, Hunt GJ (2012). Fine-scale linkage mapping reveals a small set of candidate genes influencing honey bee grooming behavior in response to Varroa mites. Plos One.

[62] Behrens D (2011). Three QTL in the honey bee *Apis mellifera* L. suppress reproduction of the parasitic mite *Varroa destructor*. Ecol. Evol..

[63] Lattorff HMG, Buchholz J, Fries I, Moritz RFA (2015). A selective sweep in a *Varroa destructor* resistant honeybee (*Apis mellifera*) population. Infect. Genet. Evol..

[64] Conlon BH (2019). A gene for resistance to the *Varroa mite* (Acari) in honey bee (*Apis mellifera*) pupae. Mol. Ecol..

[65] Conlon BH (2018). The role of epistatic interactions underpinning resistance to parasitic *Varroa mites* in haploid honey bee (*Apis mellifera*) drones. J. Evol. Biol..

[66] Broeckx BJG (2019). Honey bee predisposition of resistance to ubiquitous mite infestations. Sci. Rep..

[67] Blacquiere T (2019). Darwinian black box selection for resistance to settled invasive *Varroa destructor* parasites in honey bees. Biol. Invas..

[68] Panziera D, Langevelde F, Blacquière T (2017). Varroa sensitive hygiene contributes to naturally selected varroa resistance in honey bees. J. Apic. Res..

[69] Kefuss J, Vanpoucke J, Bolt M, Kefuss C (2015). Selection for resistance to *Varroa destructor* under commercial beekeeping conditions. J. Apic. Res..

[70] Chandler CH, Chari S, Dworkin I (2013). Does your gene need a background check? How genetic background impacts the analysis of mutations, genes, and evolution. Trends Genet..

[71] Nadeau JH (2001). Modifier genes in mice and humans. Nat. Rev. Genet..

[72] Chow CY (2016). Bringing genetic background into focus. Nat. Rev. Genet..

[73] Dowell RD (2010). Genotype to phenotype: A complex problem. Science.

[74] Hummel KP, Coleman DL, Lane PW (1972). The influence of genetic background on expression of mutations at the diabetes locus in the mouse. I. C57BL-KsJ and C57BL-6J strains. Biochem. Genet..

[75] Kruitwagen A, van Langevelde F, van Dooremalen C, Blacquiere T (2017). Naturally selected honey bee (*Apis*
*mellifera*) colonies resistant to *Varroa destructor* do not groom more intensively. J. Apic. Res..

[76] Free JB (1988). New Order for the Apiary.

[77] Ruttner F (2013). Biogeography and Taxonomy of Honeybees.

[78] Canovas F, De la Rua P, Serrano J, Galian J (2008). Geographical patterns of mitochondrial DNA variation in *Apis*
*mellifera*
*iberiensis* (Hymenoptera: Apidae). J. Zool. Syst. Evol. Res..

[79] Chávez-Galarza J (2017). Mitochondrial DNA variation of *Apis*
*mellifera*
*iberiensis*: Further insights from a large-scale study using sequence data of the tRNA^leu^-cox2 intergenic region. Apidologie.

[80] Tihelka E, Cai CY, Pisani D, Donoghue PCJ (2020). Mitochondrial genomes illuminate the evolutionary history of the Western honey bee (*Apis*
*mellifera*). Sci. Rep..

[81] Calderon RA, Sommeijer MJ, de Ruijter A, van Veen JW (2003). The reproductive ability of *Varroa destructor* in worker brood of Africanized and hybrid honey bees in Costa Rica. J. Apic. Res..

[82] Elmi M, Rafat SA, Alijani S, Tahmasbi G, Javanmard A (2021). Expression of suppression of mite reproduction in drone brood cells of honey bees of different genotypic groups in East Azarbaijan Province of Iran. Iran. J. Appl. Anim. Sci..

[83] Odemer R (2020). Reproductive capacity of *Varroa destructor* in four different honey bee subspecies. Saudi J. Biol. Sci..

[84] Garrido C, Rosenkranz P, Paxton RJ, Goncalves LS (2003). Temporal changes in *Varroa destructor* fertility and haplotype in Brazil. Apidologie.

[85] Jones JC (2020). Tool for genomic selection and breeding to evolutionary adaptation: Development of a 100K single nucleotide polymorphism array for the honey bee. Ecol. Evol..

[86] Bubnic J, Mole K, Presern J, Moskric A (2020). Non-destructive genotyping of honeybee queens to support selection and breeding. Insects.

[87] Bernstein R (2023). First large-scale genomic prediction in the honey bee. Heredity.

