# Supplementary material for: Population-wide modelling reveals prospects of marker-assisted selection for parasitic mite resistance in honey bees
Source: Sci Rep. 2024 Apr 3;14:7866. doi: 10.1038/s41598-024-58596-5 (PMC10991324; doi:10.1038/s41598-024-58596-5)
Supplement: Supplementary file 2 — Supplementary Information 2. [file 41598_2024_58596_MOESM2_ESM.pdf]

S1 Table. Locations of the tested variants on the Amel_HAv3.1 reference genome. Linkage groups (LG), positions and gene symbols refer to reference genome Amel_HAv3.1. SNP numbers have been allocated in accordance with[1].

SNP	LG	Position of variant on LG	Gene symbol	Gene name	Variant
**1**	LG1	24214694	LOC412088	Mucin-12 isoform X1	C>T
**2**	LG1	24214744	LOC412088	Mucin-12 isoform X1	T>C
**3**	LG3	11807235	LOC724886	Uncharacterized protein LOC724886 isoform X2	G>A
**4**	LG9	11542136	LOC100578770	Uncharacterized protein LOC100578770	T>C
**5**	LG9	11461121	LOC411744	Spectrin beta chain isoform X1	A>C
**6**	LG10	6509050	LOC408302	Solute carrier family 22 member 21	C>T
**7**	LG15	4695209	LOC410626	Sodium-coupled monocarboxylate transporter 1	C>T
**8**	LG15	5701853	LOC551562	Dynein beta chain, ciliary	T>C

[1] Bouuaert, D.C., et al., *qPCR assays with dual-labeled probes for genotyping honey bee variants associated with varroa resistance*. Bmc Veterinary Research, 2021. 17(1).
