# Supplementary material for: Population-wide modelling reveals prospects of marker-assisted selection for parasitic mite resistance in honey bees
Source: Sci Rep. 2024 Apr 3;14:7866. doi: 10.1038/s41598-024-58596-5 (PMC10991324; doi:10.1038/s41598-024-58596-5)
Supplement: Supplementary file 3 — Supplementary Information 3. [file 41598_2024_58596_MOESM3_ESM.pdf]

SNP1

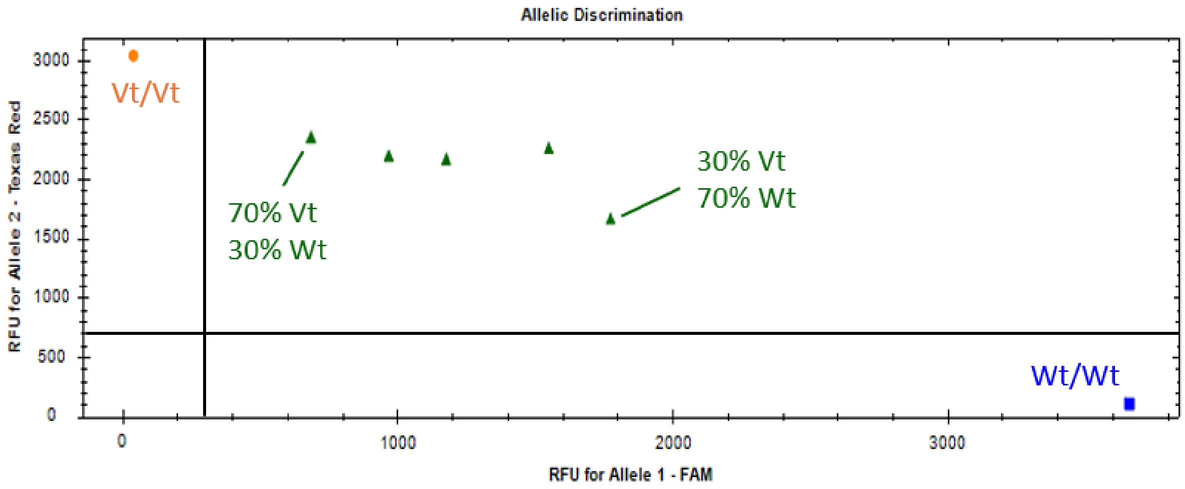

SNP2

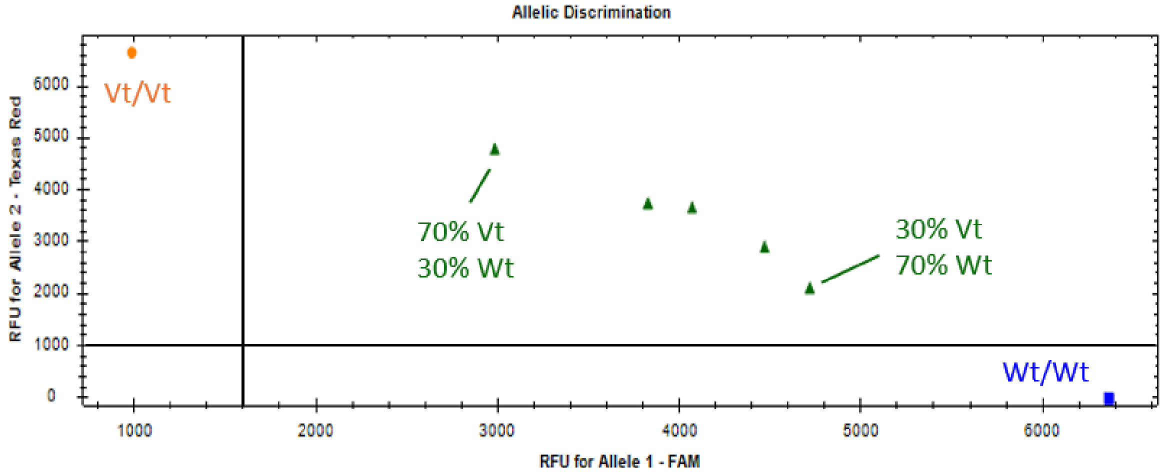

SNP3

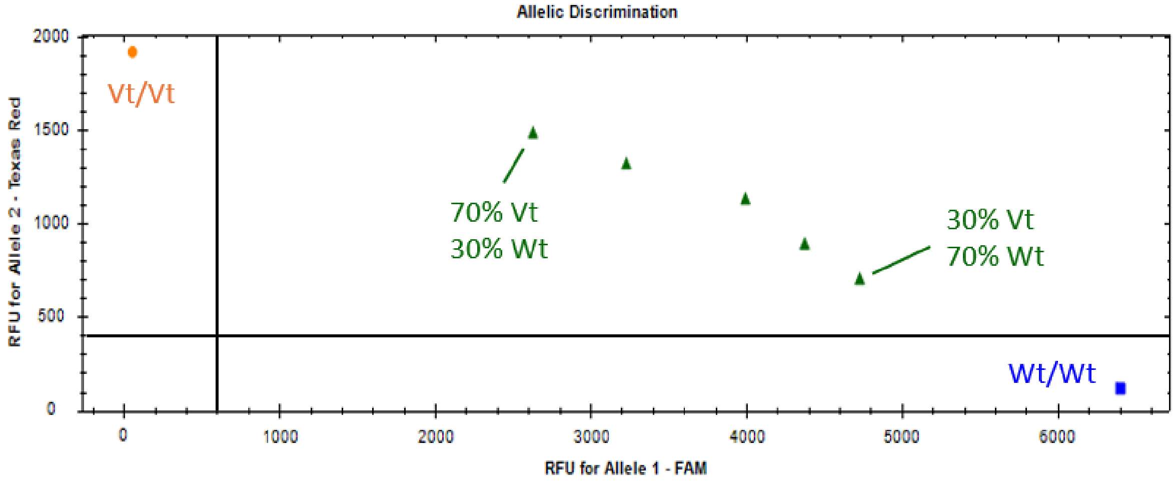

SNP4

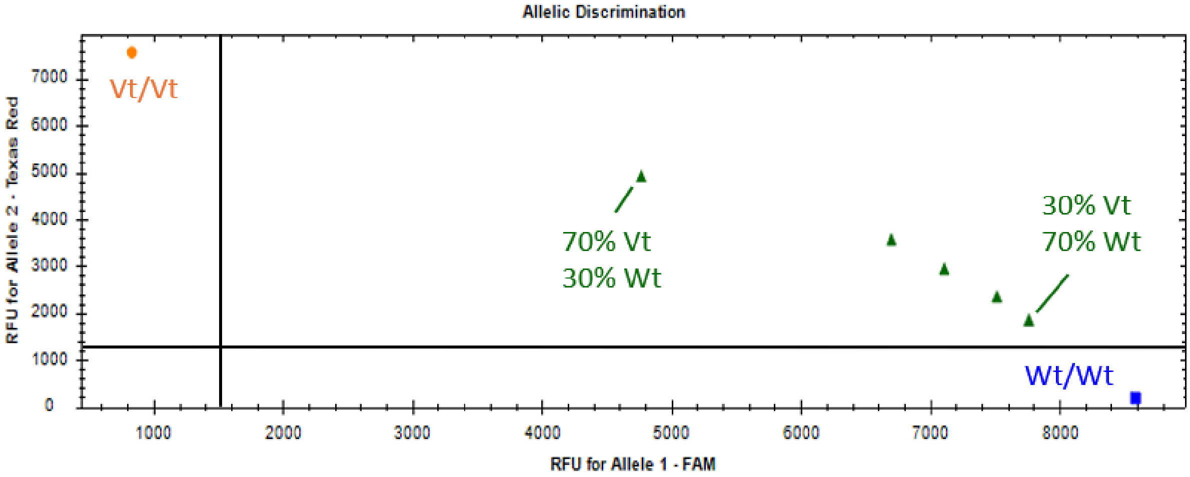

SNP5

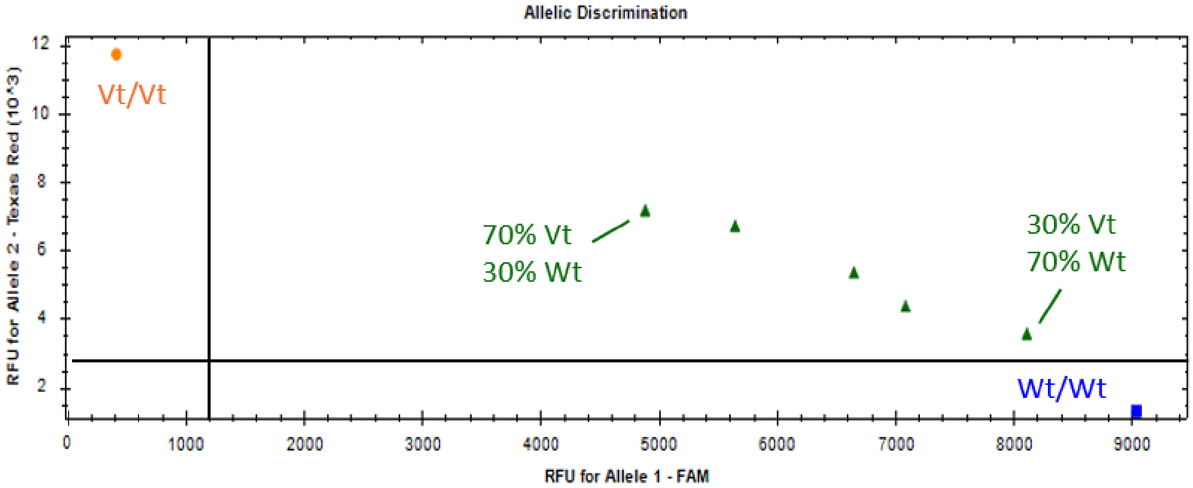

SNP6

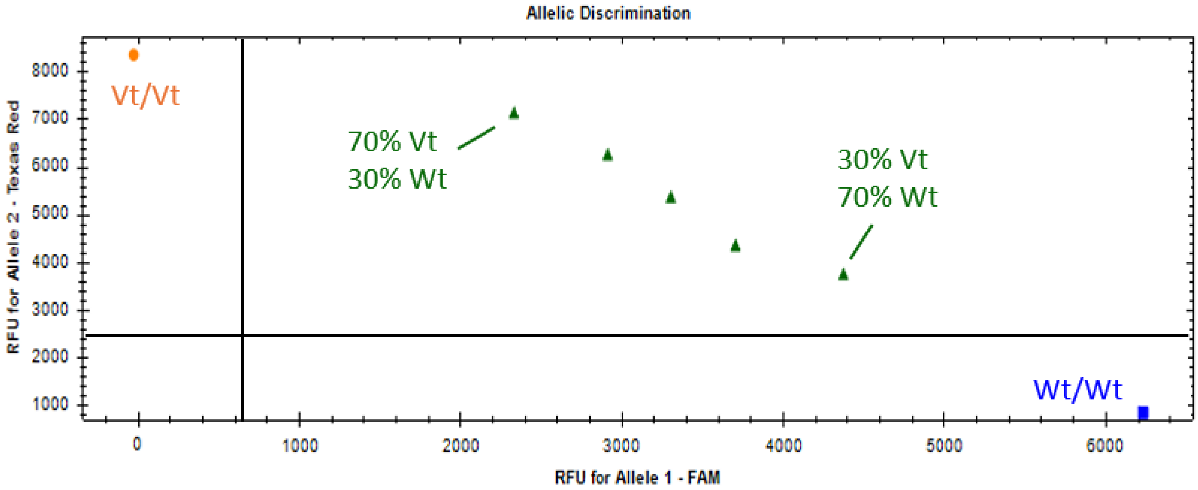

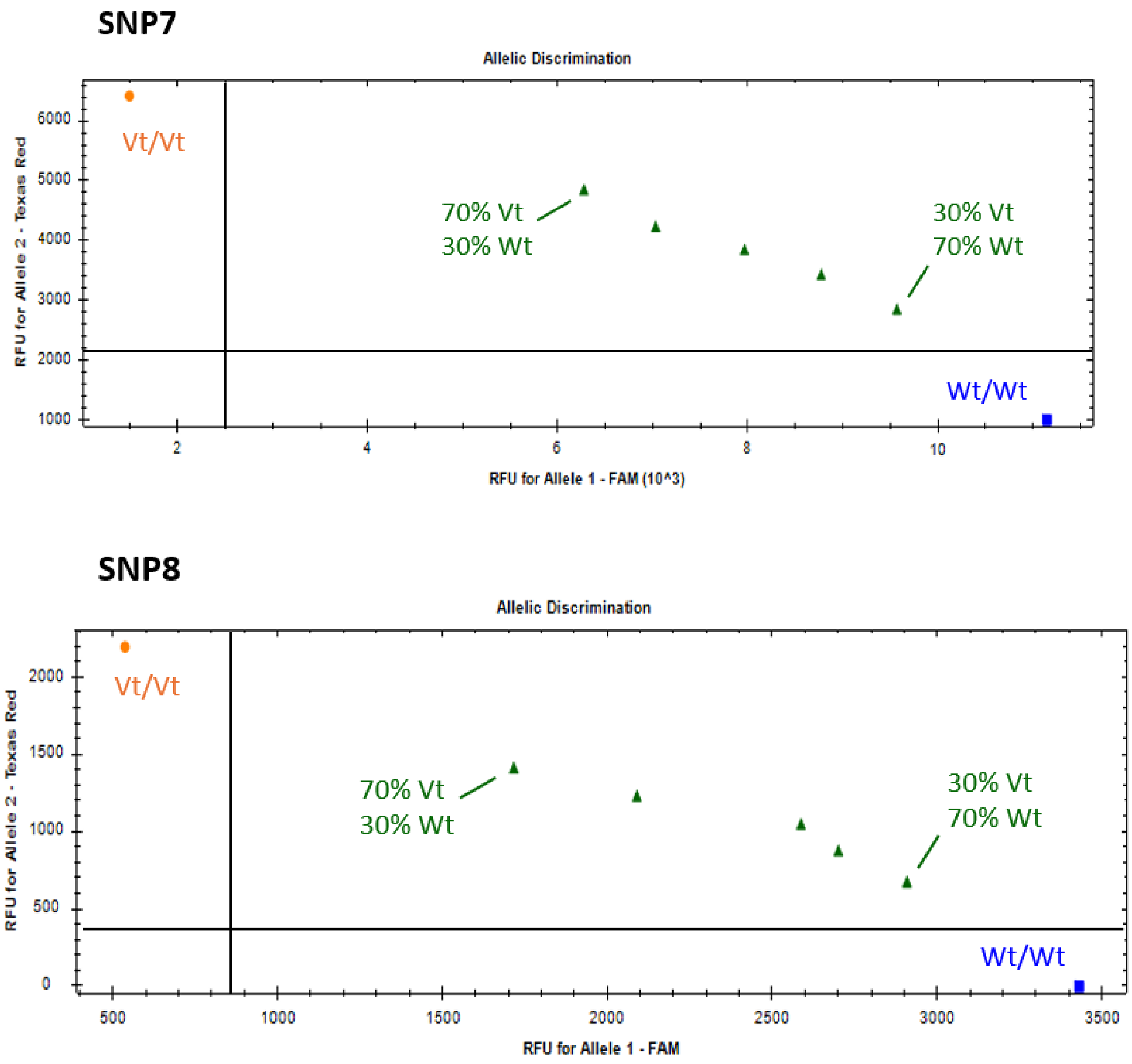

Figure S2. Lab tests with post-genotyped pooled drones showed that proportions of wild-type and variant-type drones in the interval of [30%-70%] are easily capable of distinguishing homozygous queens from heterozygous queens for a specific SNP of interest. More specifically, we genotyped multiple drones individually (thorax) with the qPCR with dual-labeled probes and pooled their legs afterwards in different proportions of wild type (Wt) and variant type (Vt) allele. After running the extracted DNA of the different proportions of pooled legs on qPCR with dual labelled probes, we could clearly distinguish homozygous wild type or - variant type queens from heterozygous queens with an allelic discrimination plot if proportions of Wt and Vt allele were in the interval [30%-70%]. SNP numbers have been allocated in accordance with[1].

[1] Bouuaert, D.C., et al., *qPCR assays with dual-labeled probes for genotyping honey bee variants associated with varroa resistance.* Bmc Veterinary Research, 2021. 17(1).
