# Supplementary material for: Population-wide modelling reveals prospects of marker-assisted selection for parasitic mite resistance in honey bees
Source: Sci Rep. 2024 Apr 3;14:7866. doi: 10.1038/s41598-024-58596-5 (PMC10991324; doi:10.1038/s41598-024-58596-5)
Supplement: Supplementary file 4 — Supplementary Information 4. [file 41598_2024_58596_MOESM4_ESM.pdf]

S2 Table. Number of distinct colony drone brood samples provided in 2020 and 2021 by each beekeeper involved in the population-wide genotype-phenotype association study.

**Beekeeper**	**2020**	**2021**	**Total**
**1**	7	6	13
**2**	7	4	11
**3**	4	7	11
**4**	2	5	7
**5**	6	1	7
**6**	1	5	6
**7**	3	3	6
**8**	0	6	6
**9**	0	5	5
**10**	1	4	5
**11**	2	3	5
**12**	2	3	5
**13**	4	1	5
**14**	3	2	5
**15**	0	5	5
**16**	3	1	4
**17**	3	1	4
**18**	1	3	4
**19**	1	3	4
**20**	3	1	4
**21**	0	3	3
**22**	1	2	3
**23**	0	3	3
**24**	0	3	3
**25**	3	0	3
**26**	2	0	2
**27**	2	0	2
**28**	2	0	2
**29**	2	0	2
**30**	0	2	2
**31**	0	2	2
**32**	0	2	2
**33**	0	1	1
**34**	0	1	1
**35**	0	1	1
**36**	0	1	1
**37**	0	1	1
**38**	0	1	1
**39**	0	1	1
**40**	0	1	1
**41**	0	1	1
**42**	0	1	1
**43**	0	1	1
**Sum**	**65**	**97**	**162**

Beekeepers are anonymized by numbers.
