# Supplementary material for: Population-wide modelling reveals prospects of marker-assisted selection for parasitic mite resistance in honey bees
Source: Sci Rep. 2024 Apr 3;14:7866. doi: 10.1038/s41598-024-58596-5 (PMC10991324; doi:10.1038/s41598-024-58596-5)
Supplement: Supplementary file 5 — Supplementary Information 5. [file 41598_2024_58596_MOESM5_ESM.pdf]

S3 Table. Number of distinct colony drone brood samples provided in 2021 and 2022 by each beekeeper involved in the allelic frequency analysis by genotyping honey bee queens from Flanders.

Beekeeper	2021	2022	Total
**1**	3	6	9
**2**	3	2	5
**3**	0	1	1
**4**	2	0	2
**5**	5	3	8
**6**	3	0	3
**7**	5	0	5
**8**	0	1	1
**9**	2	0	2
**10**	5	2	7
**11**	12	12	24
**12**	0	1	1
**13**	5	0	5
**14**	3	3	6
**15**	0	1	1
**16**	4	8	12
**17**	4	4	8
**18**	0	1	1
**19**	2	0	2
**20**	4	0	4
**21**	1	4	5
**22**	1	0	1
**23**	2	0	2
**24**	2	0	2
**25**	3	0	3
**26**	0	5	5
**27**	0	2	2
**28**	0	2	2
**29**	11	0	11
**30**	4	0	4
**31**	1	1	2
**32**	2	0	2
**33**	0	1	1
**34**	9	0	9
**35**	1	0	1
**36**	3	2	5
**37**	1	4	5
**38**	6	0	6
**39**	3	0	3
**40**	6	0	6
**41**	3	3	6
**42**	2	0	2
**43**	1	0	1
**44**	6	8	14
**45**	6	5	11
**46**	7	5	12
**47**	0	3	3
**48**	0	2	2
**49**	3	7	10
**50**	9	10	19
**51**	5	0	5

**52**	4	5	9
**53**	3	0	3
**54**	4	7	11
**Sum**	**171**	**121**	**292**

Beekeepers are anonymized by numbers. Beekeeper numbers do not match with the numbers in S2 Table.
